# Integrin α5β1-fimbriae binding and actin rearrangement are essential for *Porphyromonas gingivalis* invasion of osteoblasts and subsequent activation of the JNK pathway

**DOI:** 10.1186/1471-2180-13-5

**Published:** 2013-01-10

**Authors:** Wenjian Zhang, Jun Ju, Todd Rigney, Gena Tribble

**Affiliations:** 1 Department of Diagnostic and Biomedical Sciences, University of Texas School of Dentistry at Houston, 7500 Cambridge Street, Suite 5366, Houston, TX, 77054, USA; 2 Department of Periodontics and Dental Hygiene, University of Texas School of Dentistry at Houston, 7500 Cambridge Street, Houston, TX, 77054, USA

**Keywords:** Osteoblasts, *Porphyromonas gingivalis*, Integrins, Cytoskeleton, Signaling, Apoptosis

## Abstract

**Background:**

Chronic periodontitis is an infectious disease of the periodontium, which includes the gingival epithelium, periodontal ligament and alveolar bone. The signature clinical feature of periodontitis is resorption of alveolar bone and subsequent tooth loss. The Gram-negative oral anaerobe, *Porphyromonas gingivalis*, is strongly associated with periodontitis, and it has been shown previously that *P. gingivalis* is capable of invading osteoblasts in a dose- and time-dependent manner resulting in inhibition of osteoblast differentiation and mineralization *in vitro*. It is not yet clear which receptors and cytoskeletal components mediate the invasive process, nor how the signaling pathways and viability of osteoblasts are affected by bacterial internalization. This study aimed to investigate these issues using an *in vitro* model system involving the inoculation of *P. gingivalis* ATCC 33277 into primary osteoblast cultures.

**Results:**

It was found that binding between *P. gingivalis* fimbriae and integrin α5β1 on osteoblasts, and subsequent peripheral condensation of actin, are essential for entry of *P. gingivalis* into osteoblasts. The JNK pathway was activated in invaded osteoblasts, and apoptosis was induced by repeated infections.

**Conclusions:**

These observations indicate that *P. gingivalis* manipulates osteoblast function to promote its initial intracellular persistence by prolonging the host cell life span prior to its intercellular dissemination via host cell lysis. The identification of molecules critical to the interaction between *P. gingivalis* and osteoblasts will facilitate the development of new therapeutic strategies for the prevention of periodontal bone loss.

## Background

*Porphyromonas gingivalis* is a Gram-negative, black-pigmented anaerobe that is recognized as one of the primary etiologic agents of adult chronic and severe periodontal disease [1]. *P. gingivalis* is able to invade gingival epithelial cells and fibroblasts and reach deeper periodontal tissues, including the surface of alveolar bone [2-4]. Previous studies from our laboratory have demonstrated the invasion of osteoblasts by *P. gingivalis* in a dose- and time-dependent manner, which results in an inhibition of osteoblast differentiation and mineralization in an in vitro repetitive inoculation system [5,6]. However, the detailed mechanism by which *P. gingivalis* invades osteoblasts, e.g., the cellular receptors and cytoskeletal proteins involved, and how the signaling pathways and viability of osteoblasts are influenced by *P. gingivalis* infection, remain unclear.

Many bacterial species, including group A streptococci [7], *Staphylococcus aureus*[8], and *Escherichia coli*[9], can exploit host receptors, particularly integrins, for adhering to and invading host cells. *P. gingivalis* has been demonstrated to adhere to and invade gingival epithelial and endothelial cells via an interaction between bacterial fimbriae and α5β1 integrins [10-12].

The host cell cytoskeleton is a downstream target of integrin signaling [13]. Bacterial invasion mediated through integrins is usually associated with minimal and transient cytoskeletal remodeling [14]. Invasion of *P. gingivalis* into gingival epithelial cells induces the nucleation of actin filaments to form microspike-like protrusions and long stable microfilaments distributed throughout the cells [15]. Cytoskeletal reorganization may facilitate phagocytic cup formation and subsequent bacterial engulfment.

Cytoskeletal remodeling resulting from bacterial internalization can spatially redistribute enzymes such as MAPK family members and their substrates, and thus influence intracellular signaling pathways [16,17]. *P. gingivalis* invasion of human gingival epithelial cells causes activation of JNK (c-Jun N-terminal kinase) and down-regulation of ERK1/2 (extracellular signal regulated kinase), whereas p38 and NF-κB (Nuclear factor-Kappa B) are not affected [18].

After invading gingival cells, *P. gingivalis* ultimately localizes to the perinuclear region [2,4]. Despite the burden of a large number of intracellular *P. gingivalis*, both gingival epithelial cells and fibroblasts demonstrate an initially decreased but later increased rate of apoptosis upon bacterial challenge [19-22]. Presumably, this temporal shift from cell survival to apoptosis is utilized by *P. gingivalis* to reach an initial intracellular concentration while escaping host immune surveillance, and a later dismantling of host cells to facilitate disease transmission.

This paper reports results from experiments using an *in vitro* model of *P. gingivalis*−osteoblast interactions. The findings suggest that *P. gingivalis* uses its major fimbriae to bind to integrin α5β1 on osteoblasts and reorganize actin microfilaments to invade osteoblasts. In addition, infected osteoblasts demonstrate activation of the JNK pathway, as well as an initial increase in cellular survival with a subsequent increased cellular death, as reported for other periodontal cells.

## Methods

### Osteoblast isolation

Primary mouse calvarial osteoblasts were isolated from 7-day-old CD-1 mice using the method described by Wong and Cohn [23]. Briefly, calvaria were subjected to four sequential 15-minute digestions in an enzyme mixture containing 0.05% trypsin and 0.1% collagenase P at 37°C. Cell fractions 2–4 were pooled and resuspended in Dulbecco’s Modified Eagle’s Medium (DMEM) containing 10% fetal bovine serum (FBS), 100 U/ml penicillin and 100 μg/ml streptomycin, then filtered through a 70 μm cell strainer. Cells were plated at a density of 1 × 10^4^ cells/cm^2^ and the medium was changed 24 h later. All animal-related experiments were approved by the Center for Laboratory Animal Medicine and Care at the University of Texas Health Science Center at Houston (approved animal protocol number HSC-AWC-10–145).

### Bacteria and culture conditions

*Porphyromonas gingivalis* strain ATCC 33277 was grown anaerobically at 37°C in a Coy anaerobic chamber under an atmosphere of 86% nitrogen, 10% carbon dioxide, 4% hydrogen. The culture medium was Trypticase Soy Broth (TSBY) supplemented with 5% yeast extract, 2% sodium bicarbonate, 7.5 μM hemin and 3 μM menadione. TSB blood agar plates (BAP) were made with the addition of 5% sheep’s blood and 1.5% agarose. The bacteria were inoculated from BAP into 5 ml of TSBY and cultured anaerobically for 18 to 24 h at 37°C, then diluted in TSBY and grown to early log phase. Bacterial cells were harvested by low-speed centrifugation and resuspended in α-MEM (alpha minimum essential medium). Bacteria were then diluted in α-MEM to generate the appropriate MOI (multiplicity of infection) for addition to osteoblast cultures.

### Bacterial inoculation

To identify the receptors utilized by *P. gingivalis* during invasion of osteoblasts, *P. gingivalis* was inoculated into 1-week-old osteoblast cultures at a MOI of 150 for 1 h. To evaluate osteoblast cytoskeleton rearrangement upon *P. gingivalis* infection, *P. gingivalis* was inoculated into 1-week-old osteoblast cultures at a MOI of 150 for 30 min, 3 h or 24 h. For signaling pathway and apoptosis assays, bacteria were inoculated at a MOI of 150 for 3 h in 1-week old osteoblast cultures (designated as day 1 on bacterial inoculation), then every other day up to day 21. For all inoculations, the osteoblasts were washed with PBS and then incubated with viable *P. gingivalis* at 37°C in 5% CO_2_/95% air for the time periods described above. Osteoblasts were washed with PBS again and cultured in fresh α-MEM until the next inoculation. Controls were subjected to the same media change and wash conditions without the addition of bacteria.

### Western blotting

Primary mouse calvarial osteoblasts were isolated and plated in 6-well plates in DMEM supplemented with 10% FBS and antibiotics. After 1 week, the medium was changed to α-MEM supplemented with 10% FBS, 50 μg/ml ascorbic acid and 4 mM β-glycerophosphate to induce the differentiation of osteoblasts. The medium was changed every other day thereafter. On each medium change day, viable *P. gingivalis* 33277 was inoculated into the cultures at a MOI of 150 for 3 h, and this procedure was carried out for 3 weeks. Protein was extracted from the cultures at the end of each week with ice-cold RIPA buffer (50 mM Tris-HCl pH 7.4, 150 mM NaCl, 1 mM ethylenediaminetetraacetic acid (EDTA), protease inhibitors (1 μg/ml leupeptin, 0.5 μg/ml pepstatin, 0.7 μg/ml aprotonin, 0.5 mM phenylmethylsulfonyl fluoride), 1% Triton X-100, 1% sodium deoxycholate, 0.1% sodium dodecyl sulfate (SDS), and 0.004% sodium azide) by shaking at 4°C for 15 min. The homogenates were centrifuged at 10,000 × g for 20 min at 4°C. The supernatant protein concentration was determined by BCA assay. Proteins (20 μg) were separated by sodium dodecyl sulfate-polyacrylamide gel electrophoresis (SDS-PAGE) on 10–20% gels and transferred to nitrocellulose membranes. The blots were incubated with rabbit anti-integrin α5 or β1 polyclonal antibody (1:500; Millipore, Temecula, CA), rabbit anti-large fragment of cleaved caspase-3 polyclonal antibody (1:100; Millipore), rabbit anti-ERK, JNK, or p38 polyclonal antibody (all 1:1000), rabbit anti-mouse phosphorylated (p-) ERK, p-JNK, p-p38 monoclonal antibody (all 1:1000; Cell Signaling Technology, Danvers, MA), and HRP-conjugated goat anti-actin polyclonal antibody (Santa Cruz Biotechnology, Santa Cruz, CA) overnight at 4°C. The blots were washed and then incubated with goat anti-rabbit HRP conjugated secondary antibody (1:10,000) for 1 h at RT. Protein bands were visualized using an Immun-Star^TM^ HRP substrate kit (BioRad, Hercules, CA). The blots were developed and scanned, and densitometric analysis was performed with Kodak 1D Image Analysis Software (Eastman Kodak, Rochester, NY).

### Immunoprecipitation

Freshly isolated osteoblasts were plated in 6-well plates in DMEM supplemented with 10% FBS and antibiotics. On day 7, *P. gingivalis* was inoculated at a MOI of 150 for 1 h. Uninfected osteoblasts were used as controls. Osteoblasts were washed with ice-cold PBS and lysed with ice-cold RIPA buffer containing freshly added protease inhibitors. The soluble fraction was collected by centrifugation at 10,000 × g for 20 min. The cell lysates were pre-cleared by incubation with protein A Sepharose beads at 4°C for 10 min on a rocker. The concentrations of the lysates were determined by BCA assay, and were then diluted to 5 mg/ml with PBS. To 500 μl of cell lysate, rat anti-mouse α5β1 monoclonal antibody (1:25; Millipore) or rabbit anti-rFimA polyclonal antibody (1:100) was added and gently mixed overnight at 4°C on a rocker. The immunocomplexes were captured by adding 100 μl of bead slurry and gently rocking overnight at 4°C. The beads were collected by pulse centrifugation and washed with ice-cold RIPA buffer. The immunocomplexes were dissociated from the beads by boiling in SDS-PAGE sample buffer for 5 min and analyzed by western blotting with rabbit anti-integrin α5 or β1 polyclonal antibody (both 1:500; Millipore) or rabbit anti-FimA polyclonal antibody (1:2000). Crude osteoblast and *P. gingivalis* extracts were included on the western blots alone as controls to identify the bands for α5, β1, and FimA.

### Confocal fluorescence microscopy

To further identify the receptors utilized by *P. gingivalis* during invasion of osteoblasts, *P. gingivalis* was inoculated into 7-day-old osteoblast cultures at a MOI of 150 for 1 h. Uninfected osteoblasts were used as controls. The cultures were washed with PBS, fixed in 2% paraformaldehyde (PFA), permeabilized with 0.1% Nonidet P-40, and blocked with 3% BSA and 1% horse serum. The cultures were further incubated with rat anti-mouse integrin α5β1 monoclonal antibody (1:100; Millipore) and rabbit anti-*P. gingivalis* FimA polyclonal antibody (1:2000) overnight at 4°C, followed by washing and incubation with Alexa Fluor 594 conjugated goat anti-rat and Alexa Fluor 488 conjugated goat anti-rabbit secondary antibodies (both 1:200; Molecular Probes, Invitrogen, Carlsbad, CA) for 1 h at room temperature (RT). Rat and rabbit IgG isotype controls were included to validate the specificity of the staining. Osteoblast nuclei were labeled with DAPI (Molecular Probes). The confocal images were captured with an Olympus FV1000 Laser Confocal microscope using Olympus Fluoview software (Olympus America Inc. Center Valley, PA). The potential binding between osteoblast integrin α5β1 and *P. gingivalis* fimbriae was indicated by the yellow fluorescence where red (α5β1) and green (fimbriae) fluorescence co-localized.

To determine whether α5β1-fimbriae binding and/or new host protein synthesis were essential for *P. gingivalis* invasion of osteoblasts, four experimental groups were set up: 1) control, osteoblasts without *P. gingivalis* inoculation; 2) osteoblasts inoculated with *P. gingivalis*; 3) osteoblasts treated with a 1:100 dilution of rat anti-mouse integrin α5β1 monoclonal antibody (Millipore) for 1 h at RT prior to bacterial inoculation; 4) osteoblasts pretreated with the protein synthesis inhibitor, cycloheximide (50 μg/ml), 1 h prior to bacterial inoculation. For groups 2, 3 and 4, osteoblasts were inoculated with *P. gingivalis* at a MOI of 150 for 30 min, 1 h and 3 h. Thereafter, the cultures were washed, fixed, permeabilized and blocked as described above. The cells were incubated with rabbit anti-*P. gingivalis* polyclonal antibody (1:4000) for 1 hr at RT, followed by washing and incubation with Alexa Fluor 488 conjugated goat anti-rabbit secondary antibody (1:200; Molecular Probes) for 1 h at RT. Osteoblast actin and nuclei were labeled with rhodamine phalloidin (Molecular Probes) and DAPI, respectively. The internalization of *P. gingivalis* into osteoblasts was determined by the localization of the bacteria within the cytoplasmic boundary of osteoblasts, as well as the close proximity of the bacteria to osteoblast nuclei. The number of osteoblasts with bacterial invasion was counted manually and expressed as the percentage of the total number of osteoblasts counted.

To determine whether actin rearrangement is required for *P. gingivalis* invasion, osteoblasts were inoculated with *P. gingivalis* at a MOI of 150 for 30 min, 3 h and 24 h with or without the addition of the actin-disrupting agent, cytochalasin D (2.5 μg/ml), for the entire infection period. Uninfected osteoblasts were used as controls. The staining process and confocal image acquisition were performed as described above. The number of osteoblasts with bacterial invasion was counted manually and expressed as the percentage of the total number of osteoblasts counted.

### TUNEL staining

*P. gingivalis-*infected osteoblast cultures were fixed with 4% PFA in PBS. The TUNEL procedure was performed with the TACS TBL kit (R&D Systems, Minneapolis, MN) according to the manufacturer’s instructions. Nuclease treatment or exclusion of TdT enzyme was used as the positive or negative control, respectively. Light microscopic examination revealed apoptotic cells as having condensed, blue-stained nuclei. Quantification of apoptotic cells was determined manually and expressed as a percentage of the total number of cells counted.

### Statistics

All experiments were repeated independently three times. Data were analyzed using Student’s *t* test to determine the significance between groups (*P ≤* 0.05).

## Results

### Binding between integrin α5β1 and fimbriae is essential for *P. gingivalis* invasion of osteoblasts

Because the association between integrins and fimbriae mediates the invasion of *P. gingivalis* into many different host cells types, we investigated whether the entry of *P. gingivalis* into osteoblasts is mediated by integrin α5β1-fimbriae interaction.

**Figure 1:**
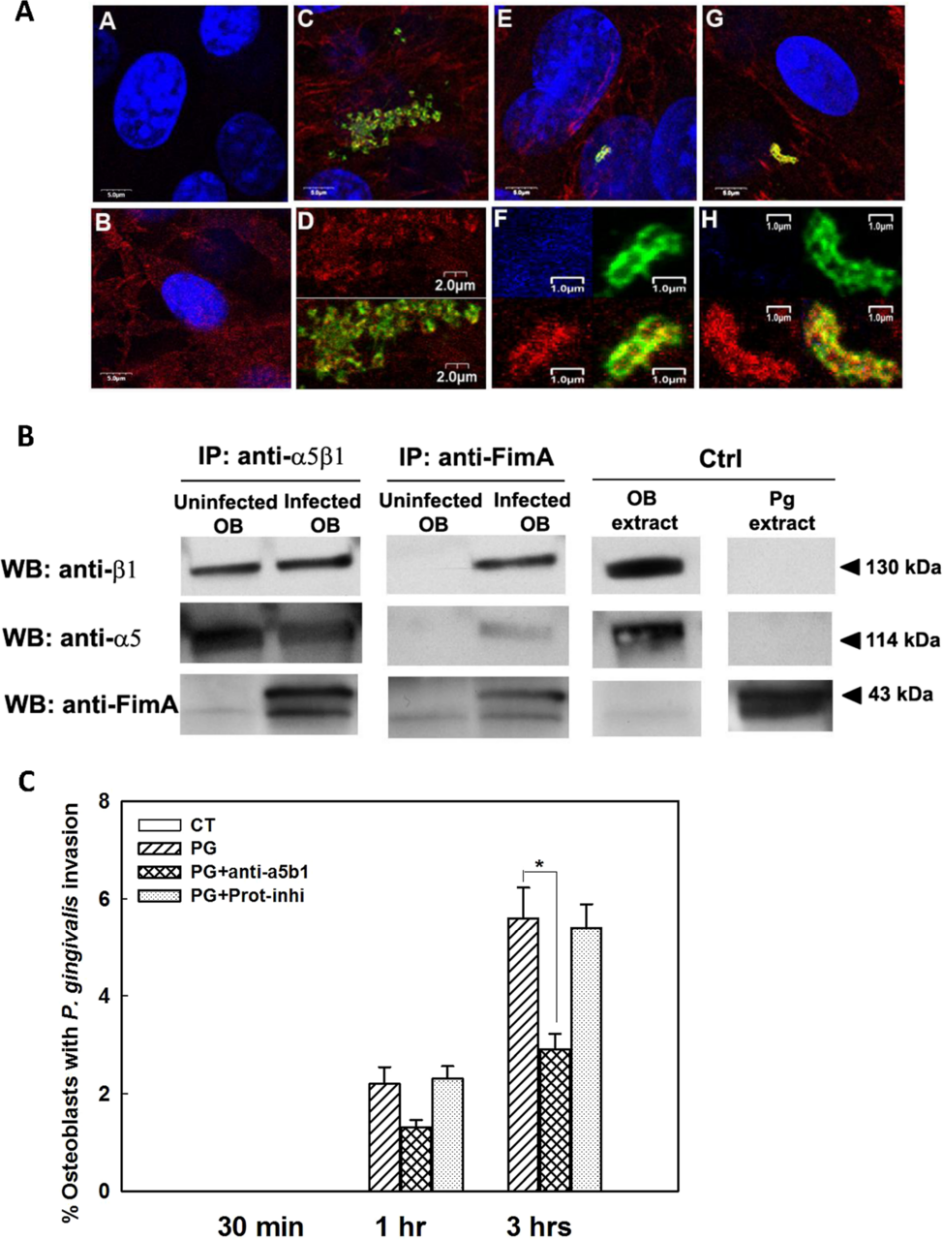
**Integrin α5β1-fimbriae binding is essential for *P. gingivalis *invasion of osteoblasts. A.** Confocal imaging demonstration of the colocalization of *P. gingivalis* fimbriae and osteoblast integrin α5β1 1 h after bacterial inoculation. Osteoblast nuclei, α5β1 integrin, and *P. gingivalis* fimbriae are labeled in blue, red and green, respectively. Panel A. Control, *P. gingivalis* was inoculated, but neither primary antibody was included. Panel B**.** Control, *P. gingivalis* was not inoculated, and both primary antibodies were included. Panels C, E and G, representative images showing the co-localization of α5β1 and fimbriae. Panels D, F and H, clipped magnified views of panels C, E and G, respectively. In panel D, the top panel shows the red channel only; the bottom panel shows the three merged channels. Panels F and H show the blue, green, and red channels and the three merged channels. Presumed binding sites are shown as yellow where the red and green labels co-localize. Note the increased red intensity at the potential binding sites. **B.** Demonstration of the physical association between integrin α5β1 and fimbriae by immunoprecipitation. Western blot showing the presence of α5 and β1 in the immunocomplex precipitated with anti-fimbriae antibody, and the presence of fimbriae in the immunocomplex precipitated with anti-α5β1 antibody in the *P. gingivalis*-infected cultures, but not in the controls. Arrowheads indicate the molecular weights of the target proteins. **C.** Association between integrin α5β1 and fimbriae is necessary for *P. gingivalis* entry into osteoblasts. Quantitative confocal imaging demonstrates that *P. gingivalis* invasion of osteoblasts is partially prevented by addition of anti-α5β1 antibody, and new α5β1 synthesis is not required for invasion. Abbreviations: IP, immunoprecipitation; Fim A, major fimbriae of P. gingivalis; Ctrl, control; OB, osteoblasts; Pg, P. gingivalis; WB, western blot, Prot-inhi, protein synthesis inhibitor; min, minute; h, hour. * denotes P < 0.05.

*P. gingivalis* fimbriae and osteoblast integrin α5β1 were labeled with green and red fluorescence, respectively. No nonspecific staining was observed in the isotype controls, indicating that the primary antibodies used were specific for their target proteins (data not shown). One hour after inoculation of *P. gingivalis* into osteoblasts cultures, cofocal imaging demonstrated many yellow regions on the surface of osteoblasts resulting from the co-localization of the red- and green-labeled antigens (Figure 1A), indicating the close proximity of or binding between integrin α5β1 and fimbriae. The red fluorescent signal was intensified where it colocalized with green signals, indicating a possible focal recruitment of integrin α5β1 where it bound *P. gingivalis* (Figure 1A).

Immunoprecipitation assays showed that integrins α5 and β1 were present in the immunocomplexes precipitated with the anti-fimbriae antibody in osteoblast cultures infected with *P. gingivalis*, but not in the control uninfected cultures. In addition, fimbriae were detected in the immunocomplexes precipitated with anti-α5β1 antibody in the infected cultures, but not in the control cultures (Figure 1B). Together with the confocal microscopy images, these results suggest that *P. gingivalis* fimbriae bind osteoblast α5β1 integrins during the invasive process.

To further investigate whether integrin α5β1-fimbriae binding is essential for *P. gingivalis* invasion of osteoblasts, anti-α5β1 antibody was added to the osteoblast cultures 1 h before the addition of bacteria. Figure 1C shows that blocking the integrin α5β1-fimbriae association significantly decreased the invasive efficiency of *P. gingivalis* 3 h after bacterial inoculation, indicating that integrin α5β1-fimbriae binding is crucial for *P. gingivalis* invasion of osteoblasts.

To determine whether the increased red fluorescence of integrins was due to increased protein expression or focal receptor recruitment, the protein synthesis inhibitor, cycloheximide, was added into the osteoblast cultures 1 h before the addition of bacteria. Figure 1C shows that inhibition of host protein synthesis did not interfere with the invasion of osteoblasts by *P. gingivalis*. Together with western blot analysis, which showed no appreciable change in integrin α5β1 expression in the osteoblast cultures 24 h after *P. gingivalis* inoculation (data not shown), these results indicate that integrin α5β1 is locally recruited to bind fimbriae and facilitates the internalization of *P. gingivalis*.

### Rearrangement of actin is required for *P. gingivalis* invasion of osteoblasts

**Figure 2:**
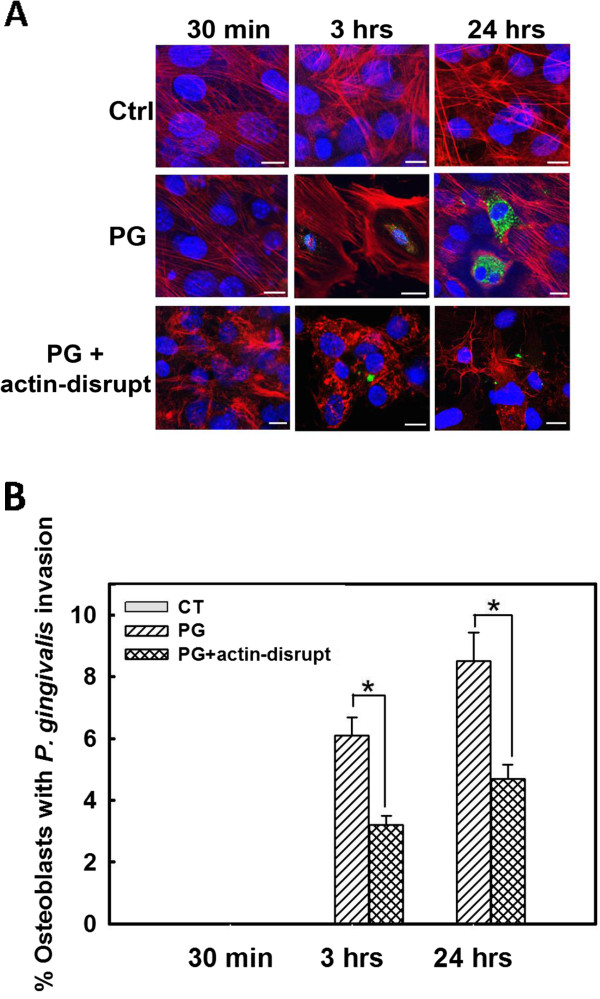
**Actin filament rearrangement is essential for *P. gingivalis *invasion of osteoblasts. A.** Osteoblast nuclei, actin and *P. gingivalis* are indicated by blue, red or green fluorescence, respectively. No appreciable change in actin filament organization was seen 30 min after infection. At 3 h, actin relocated to the periphery of the osteoblasts, leaving a void space surrounding the osteoblast nuclei occupied by *P. gingivalis*. Twenty-four hours after infection, actin became more condensed and formed a cortical outer shell. The number of perinuclear *P. gingivalis* was also significantly increased. Addition of the actin disrupting agent, cytochalasin D, reduced the number of osteoblasts with *P. gingivalis* invasion. Notice that actin had now become disorganized, as demonstrated by the punctuated pattern. **B.** Quantitative analysis of confocal images demonstrated that *P. gingivalis* invasion of osteoblasts was inhibited by the disruption of actin filaments. Abbreviations: min, minute; h, hour; Ctrl and CT, control, non-infected osteoblasts; PG, *P. gingivalis*. Scale bar = 20 μm. * denotes P < 0.05.

*P. gingivalis* was inoculated into osteoblast cultures for 30 min, 3 h or 24 h. Osteoblast nuclei, osteoblast actin, and *P. gingivalis* were labeled with blue, red, and green fluorescence, respectively, and analyzed by confocal microscopy. Compared with uninfected control cells, there was no noticeable change in actin assembly in *P. gingivalis* infected osteoblasts 30 min after inoculation. Three hours after bacterial inoculation, many osteoblasts demonstrated peripheral shifting of actin, resulting in a void space between the nuclei and cell membrane occupied with intracellular *P. gingivalis*. Actin became more concentrated and formed a cortical “shell” surrounding invaded osteoblasts 24 h after infection, and the number of perinuclear *P. gingivalis* increased significantly (Figure 2A). No appreciable change in microtubules was observed in *P. gingivalis* infected osteoblasts at any of the experimental time points (data not shown).

To investigate whether actin rearrangement is necessary for *P. gingivalis* entry into osteoblasts, the actin-disrupting agent cytochalasin D was added to the cultures together with the bacteria. Figure 2A shows that cytochalasin D treated osteoblasts demonstrated disorganized and punctuated actin filaments. Quantitative image analysis demonstrated that the bacterial invasion of osteoblasts was significantly less following treatment with cytochalasin D compared with untreated cells (Figure 2B), indicating that actin rearrangement is essential for *P. gingivalis* invasion of osteoblasts.

### The JNK pathway is activated in osteoblasts upon repeated infection with *P. gingivalis*

**Figure 3:**
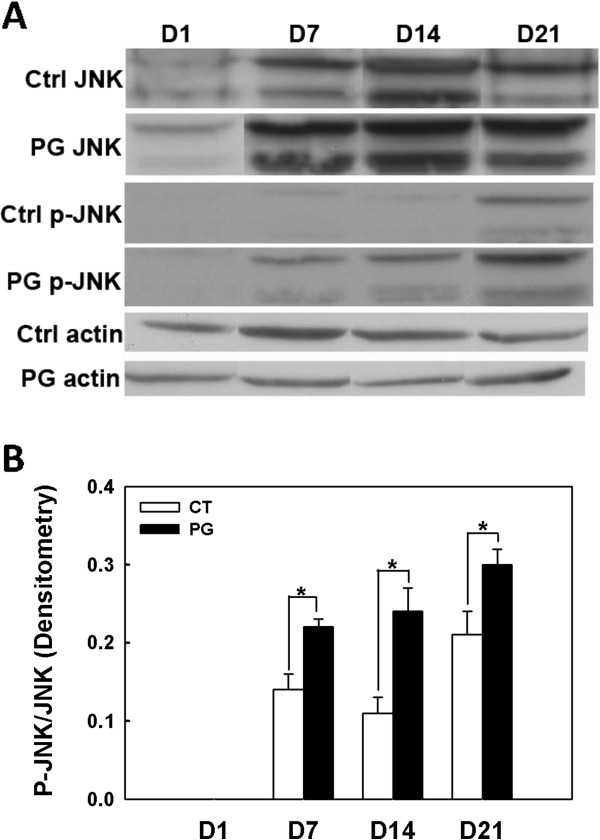
**JNK pathway is activated in osteoblasts upon repeated *P. gingivalis *infection. A.***P. gingivalis* infected osteoblasts demonstrated stronger expression of phosphorylated JNK than uninfected control cells from day 7 to 21. Actin is the loading control. **B.** Quantitative densitometric analysis showing that the p-JNK/JNK ratio was significantly higher in infected osteoblasts compared with control cells. Abbreviations: PG, *P. gingivalis*; Ctrl, control, non-infected osteoblasts; D, day; JNK, c-Jun N-terminal kinase; p-, phosphorylated. * denotes P < 0.05.

Because the MAPK pathway is activated by many host-pathogen interactions, we investigated whether this pathway is activated in osteoblasts infected with *P. gingivalis*. Considering that periodontitis is a chronic infectious disease, we inoculated *P. gingivalis* into osteoblast cultures repeatedly every other day for up to 3 weeks to mimic the chronic nature of this disease. Western blot analysis showed that phosphorylated JNK (p-JNK) bands were more intense in treated cells than in control cells from day 7 to day 21 (Figure 3A), whereas there was no noticeable change in ERK and p38 (data not shown). After normalization to actin, quantitative densitometric analysis showed that the p-JNK/JNK ratio was significantly higher in the infected osteoblasts compared with control cells (Figure 3B), indicating that the JNK pathway was activated in osteoblasts chronically infected with *P. gingivalis*.

### *P. gingivalis* initially suppresses but later promotes apoptosis in osteoblasts

**Figure 4:**
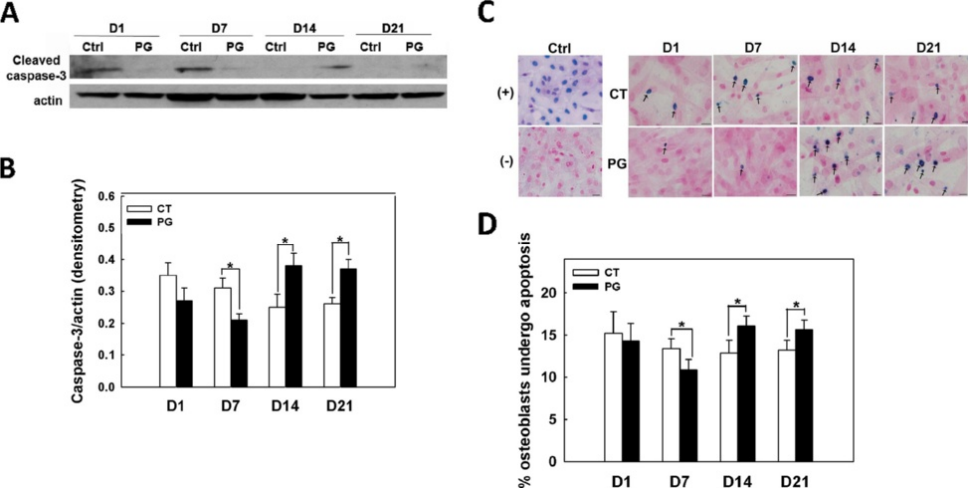
***P. gingivalis *initially suppresses but later promotes osteoblast apoptosis. A.** Western blot demonstrating the initial weak (day 1–7) but later more intense (day 14–21) cleaved caspase-3 expression in *P. gingivalis*-infected osteoblasts compared with uninfected control cells. Actin is the loading control. **B.** Quantitative densitometric analysis of cleaved caspase-3 by western blotting. Abbreviations: Ctrl, non-infected osteoblasts; PG, *P. gingivalis* infected osteoblasts; D, day. **C.** TUNEL assay showing significantly less apoptotic osteoblasts from day 1–7, followed by significantly more apoptotic osteoblasts from day 14–21 in *P. gingivalis*-infected cultures compared with uninfected control cells. Arrows denote condensed, blue-stained, apoptotic osteoblast nuclei. Nuclease treatment and exclusion of TdT enzyme were used as positive and negative controls, respectively. **D.** Quantitative analysis of the TUNEL assay data. Abbreviations: (+) Ctrl, nuclease treated, used as positive technique control; (-) Ctrl, exclusion of TdT enzyme, used as negative technique control; CT, uninfected osteoblasts; PG, *P. gingivalis* infected osteoblasts; D, day. Scale bar = 20 μm. * denotes P < 0.05.

To determine whether osteoblast viability is affected by chronic *P. gingivalis* infection, total protein was extracted from *P. gingivalis*-infected and control cultures, and western blotting was performed to detect the large fragment of cleaved caspase-3, which is an indication of the activation of apoptotic pathways. Figure 4A shows that the cleaved caspase-3 bands were weak from day 1 to day 7, but became more intense from day 14 to day 21 in the infected cultures compared with controls. This observation was validated by densitometric analysis as shown in Figure 4B, which suggests an initial suppression, but a later promotion of osteoblast apoptosis by *P. gingivalis*. Furthermore, TUNEL staining demonstrated significantly fewer condensed, blue-stained apoptotic osteoblast nuclei in the infected cultures in the first week, but significantly more apoptotic nuclei in the last two weeks of infected culture compared with control cells (Figure 4C). Again, this observation was supported by the quantitative analysis, which demonstrated a shift in the cell death pattern in the infected osteoblast cultures compared with control cells (Figure 4D).

## Discussion

In this study, we investigated both the short-term and long-term interactions between *P. gingivalis* and osteoblasts. Processes important for invasion, including engagement of receptors and rearrangement of the cytoskeleton, were examined in a narrow time window (30 min, 1 h, 3 h, and 24 h). The influence of bacterial infection on osteoblast signaling and viability was investigated over a broad time frame of 3 weeks after initial bacterial inoculation.

Our results demonstrate that *P. gingivalis* fimbriae bind osteoblast integrin α5β1 during the invasive process. Because *P. gingivalis* also exploits integrin α5β1 to enter gingival epithelial cells and fibroblasts [10-12], it appears that integrin α5β1 is a universal receptor for *P. gingivalis* invasion of periodontal tissues. Fimbriae-deficient *P. gingivalis* mutants still possess the residual ability to invade gingival epithelial cells [15] and osteoblasts [5], and anti-integrin α5β1 antibody does not completely block the invasion of osteoblasts by *P. gingivalis*, indicating the presence of additional, unidentified adhesins for *P. gingivalis* invasion. Future effort should be directed to identify these novel receptors to gain a full understanding of *P. gingivalis*-host interactions.

Confocal microscopy demonstrated an intensified focal signal for integrin α5β1 at the fimbriae binding sites 1 h after infection. This is consistent with studies in HeLa cells, in which integrin α5β1 was found to concentrate at the entry site of fluorescent beads coated with *P. gingivalis* membrane vesicles [11]. The invasion efficiency of *P. gingivalis* was not affected by inhibiting host protein synthesis, and western blotting showed no change in integrin α5β1 expression in osteoblasts 24 h after bacterial inoculation, suggesting that integrins are locally recruited to the bacterial binding sites to facilitate the invasion process. In another *in vitro* study, no change in integrin α3 and β1 expression was detected by western blotting 1 h after *P. gingivalis* inoculation into primary human osteoblast cultures [24].

In our study, *P. gingivalis* invasion caused rearrangement and peripheral concentration of actin filaments with no appreciable change in microtubule morphology in osteoblasts 24 h after bacterial inoculation. Other studies demonstrated remarkable disassembly and nucleation of the actin and microtubule filamentous networks in gingival epithelial cells 24 h after *P. gingivalis* infection*,* although microtubule rearrangement was less dramatic than actin rearrangement [15]. The actin disrupting agent cytochalasin D was found to profoundly prevent the invasion of osteoblasts by *P. gingivalis*, indicating that actin rearrangement is crucial for *P. gingivalis* entry into osteoblasts. It has been shown that microtubule dynamics can occur rapidly, and may not be observed by a single technique [25]. Investigations with more sophisticated technology and additional time points may be necessary to reveal the whole spectrum of microtubule dynamics in osteoblasts upon *P. gingivalis* invasion.

MAPKs play a central role in transmitting diverse extracellular stimuli to the nucleus, and are frequently associated with the invasive process of many pathogenic bacteria [26,27]. *P. gingivalis* can specifically activate JNK and down-regulate ERK1/2 in human gingival epithelial cells [18], whereas in gingival fibroblasts, the ERK1/2 pathway is activated [28]. Our study demonstrated the activation of JNK with no noticeable changes in the ERK1/2 and p38 pathways in osteoblasts after repeated *P. gingivalis* inoculation. *P. gingivalis* inhibits osteoblast differentiation and mineralization, partially via inhibition of the transcription factors, Cbfa-1 and osterix [6]. It is not clear whether the JNK pathway is also involved in this inhibitory process, because JNK seems to be able to both up- and down-regulate osteoblast differentiation [29,30].

The effect of *P. gingivalis* on osteoblast viability is similar to its effects on gingival epithelial cells and fibroblasts, in that all three types of periodontal cells demonstrate an initially decreased, but later increased, rate of programmed cell death [19,21,22]. There was an initial increased rate of apoptosis in the control uninfected cultures, which may reflect the response of newly isolated osteoblasts to *in vitro* culture conditions. In our study, *P. gingivalis* was repeatedly inoculated into osteoblast cultures, and it is therefore difficult to assess how long each individual bacterium can survive in an intracellular environment. A one-time inoculation of the bacteria into osteoblast cultures followed by antibiotic protection assay at different time points may provide more insight. The apoptotic response of the infected cultures suggests a long evolutionary relationship between *P. gingivalis* and periodontal cells, which results in a balanced association, whereby the organism first promotes its intracellular replication and persistence by sustaining the viability of host cells, and later shifts toward bacterial propagation and disease dissemination resulting from lysis of the host cells.

## Conclusions

We have demonstrated that integrin α5β1-fimbriae binding and actin rearrangement are essential for *P. gingivalis* invasion of osteoblasts in an *in vitro* infection system. Repeated bacterial inoculations cause JNK pathway activation, and the initial suppression but later promotion of osteoblast apoptosis. This study contributes to a better understanding of the pathogenic mechanism underlying periodontal disease by revealing how osteoblasts interact with *P. gingivalis* in a disease model.

## Abbreviations

α-MEM: Alpha minimum essential medium; BAP: Blood agar plates; DMEM: Dulbecco’s modified Eagle’s medium; EDTA: Ethylenediaminetetraacetic acid; ERK: Extracellular signal regulated kinase; FBS: Fetal bovine serum; Fim A: Major fimbriae; MOI: Multiplicity of infection; NF-κB: Nuclear factor- kappa B; PFA: Paraformaldehyde; *P. gingivalis*: *Porphyromonas gingivalis*; SDS-PAGE: Sodium dodecyl sulfate-polyacrylamide gel electrophoresis; TSBY: Trypticase Soy Broth.

## Competing interests

The authors declare that they have no competing interests.

## Authors’ contributions

WZ conceived the study, supervised the experiments, and drafted the manuscript. JJ carried out the osteoblast culture and molecular and cellular studies, and maintained the animal colonies. TR performed all bacteria-related studies. GT participated in the study design and critically revised the manuscript. All authors read and approved the final manuscript.
